# Developing and validating the Japanese version of professional attitude scale for nurses

**DOI:** 10.1111/inr.12627

**Published:** 2020-10-12

**Authors:** N. Takada, K. Asakura, S. Sugiyama

**Affiliations:** ^1^ Graduate School of Medicine Tohoku University Sendai Japan

**Keywords:** attitudes, instrument validation study, Japanese, nurses, nursing education, professionalism, scale development, trait approach

## Abstract

**Aim:**

We developed and psychometrically tested the Japanese version of the Professional Attitude Scale for Nurses (PASN‐J).

**Background:**

Nurses must recognize the importance of their professionalism; therefore, it is critical to quantitatively measure nurses’ professional attitudes.

**Introduction:**

This instrument validation study was designed to generate an itemized scale and examine its content validity/psychometric testing using a sample of Japanese nurses.

**Methods:**

Based on a trait approach focusing on the characteristic traits of the nursing profession, a 59‐item draft scale was generated. During November 2017, 2657 nurses from 29 facilities in Japan were surveyed. The questionnaire included demographics, the 59‐item draft scale, and a self‐report scale of nurses’ professional behaviour and nursing practice ability. Using exploratory and confirmatory factor analyses, we evaluated the construct, criterion‐related, concurrent, and known‐groups validity, and reliability of the PASN‐J.

**Results:**

Data from 1716 participants were analysed. The analyses yielded a 38‐item, 3‐factor scale that adequately fit the data. PASN‐J scores were positively correlated with nurses’ professional behaviour and nursing practice ability.

**Conclusion:**

The 38‐item PASN‐J has good reliability and validity, making it useful for measuring the current condition of nursing professionalism and evaluating nursing education. Implications for Nursing and Health Policy: This scale can evaluate nursing education and promote nurses’ professionalism. The PASN‐J will help identifying the elements of undergraduate nursing education that require further emphasis. Additionally, the PASN‐J could facilitate the development of nursing policies to promote professional development in nurses. Ultimately, evaluating nursing education with the PASN‐J enhances nurses’ professional attitudes and subsequently improves their quality of nursing, nursing efficiency and patient outcomes.

## Introduction

To establish nursing as a profession based on specialized knowledge and skills, it is first necessary for nurses themselves to recognize their professionalism. Problematically, nursing as an occupation has not been appraised as a full profession (Asakura et al. 2016; Morris‐Thompson et al. 2011). In the past, several sociologists have argued that professionalizing nurses was a difficult task (Etzioni 1964; Freidson 1970). While nurses have gradually come to be viewed as professionals, young people in general still regard nursing as a low‐status occupation that requires less intelligence and is suited for women in particular rather than as a profession requiring particular knowledge and skills (Gill & Baker, 2019; Glerean et al. 2017). To establish nursing as a profession, nurses must rectify the invalid common mindset that nurses are not smart. To accomplish this, it is meaningful to measure nurses’ professional attitudes quantitatively.

The Behavioral Inventory for Professionalism in Nursing and the Professional Values Scale were developed to measure nurses’ professionalism at the behavioural level (Weis & Schank, 2009), and several studies have employed these behavioural measures (Bijani et al. 2019; Tanaka et al. 2016). However, these approaches only provide an indicator of actualized behaviour. On the other hand, measuring professionalism at the cognitive level implies measuring potential professionalism at the behavioural level. Professionalism scales at the cognitive level have only been developed for nursing students and not for clinical nurses (Hisar et al. 2010).

However, it is vital to capture nurses’ professionalism at the cognitive level as well, especially in countries with rather conservative cultures (e.g., Japan), where behaviours can be restricted by environmental factors such as nurses’ relationship with physicians, organizational control and institutional framework. For example, while autonomy is a key element in nursing, it is still limited for nurses by physician‐led medical systems and organizational supervision. Nurses’ low status is perceived to be associated with the similarly low societal status of women (Crisp & Iro, 2018), which is consistent with the context within Japan (Sakashita 2018). Japan’s overall ranking in the 2018 Global Gender Gap Report was 110th out of 149 countries, and women’s enrolment in tertiary education and participation in the professional/technical workforce also ranked outside the top 100 countries (World Economic Forum 2018). The findings from this report indicate that the occupational authority of nurses, a predominantly female profession, is low.

Improving the professional attitudes of nurses is an element that enhances the quality of medical care and is an important international issue. The higher the nurse’s education level, the higher the professional values of the nurse and hospitals with more highly educated nurses show better patient outcomes (Aiken 2003; Poorchangizi 2019). These findings suggest that the professional level of nurses is relevant to the patient. Being able to measure the professional attitudes of nurses at a cognitive level without being bound by the behavioural constraints of the system or culture may contribute to international standardization of education that enhances the professional attitudes of nurses.

The current study aimed to develop a scale to measure nurses’ professional attitudes at the cognitive level. Attitudes consist of cognitive, affective and behavioural components (Rosenberg 1960). Since cognition predicts future behaviour, it is reasonable to presume that nurses with highly professional cognition are more likely to behave as professionals (Kraus 1995). Measuring professionalism at the cognitive level can therefore be thought of as measuring potential professionalism at the behavioural level. Because it is free from the restrictions of environmental constraints, measuring professionalism at the cognitive level may be more accurate than measuring it at the behavioural level. Our ultimate research question was thus how the notion of the professional attitudes of nurses can be redefined and how a scale that measures professional attitudes at the cognitive level can be developed.

We developed the Japanese version of the Professional Attitude Scale for Nurses (PASN‐J) and sought to verify its validity and reliability. This instrument validation study was designed to generate an itemized scale and examine its content validity/psychometric testing using a sample of Japanese nurses.

## Theoretical background

In this study, the trait approach was used to enumerate professional traits (e.g., expertise and autonomy) and estimate nurses’ degree of professionalism. The trait approach is a taxonomic approach that considers professions as distinct from other occupations in that they are seen as possessing a diverse range of differentiating characteristics (Saks 2016; Uzawa 2016). Many sociologists have discussed the traits that professionals should have (Brante 2011). Although there are minor ideological differences among the researchers, it is generally agreed that professional traits include (1) structured advanced knowledge, (2) aspirations for the public and (3) autonomy. We conceptualized nurses’ professional attitudes within the framework of these three theoretical components and hypothesized that they can be further subdivided into 12 domains.

Structured advanced knowledge was assumed to contain the following four domains: establishment of systematic knowledge, self‐growth, creation of knowledge and advancement of education. Aspirations for the public were assumed to contain the following four domains: adherence to an ethical code, participation in one’s professional community, task orientation and responsibility to fulfil requests. Autonomy was assumed to contain the following four domains: job‐related independence, autonomous clinical judgment, control over working conditions and respect for one’s profession.

## Methods

This study was designed for scale development (Appendix 1).

### Item generation

The PASN‐J was conceptually based on the three professional traits – structured advanced knowledge, aspirations for the public and autonomy – and the 12 domains mentioned earlier. Based on the three professional traits, we generated an initial item pool that specifically explored the nurse's professional attitudes by a deductive approach. These items were generated with reference to the leading relevant scale (Asakura et al. 2016; Hisar et al. 2010; Weis & Schank, 2009). Items were generated with consideration given to cognitive rather than behavioural expressions. The initial item pool consisted of 68 items. Respondents answered each item using a five‐point Likert scale from 1 (disagree) to 5 (agree).

To confirm the content validity, the items were revised and verified several times by one researcher with a doctorate and four researchers with master's degrees who were conducting research on nursing management research. The face validity was verified by pretesting the scale with 8 preliminary samples, and several items were deleted due to concerns about ceiling effect or lack of clarity. After item testing, the final item pool consisted of 59 items (Appendix 2).

### Participants and sample size

Larger sample sizes increase the generalizability of conclusions reached by means of factor analysis (DeVellis 2017). An acceptable ‘observations to variables’ ratio is 20:1 (Morgado et al. 2017); since our draft scale had 59 items, the required sample size was 1180 nurses. Since the response rate was expected to be about 50%, we distributed the questionnaire to approximately twice as many as nurses. The selection criteria for this study included registered nurses (including nursing managers). However, we excluded licenced practical nurses from this study because their professionalism is distinguished from that of registered nurses.

### Participants and setting

Concerning the setting, 65 hospitals were randomly selected from 140 hospitals located in Prefecture A in Japan. Research cooperation was requested via letters sent to the nurse manager of each selected hospital. The nursing manager then replied to the researcher stating how many questionnaires they needed at their facility. Altogether, 29 managers replied, and we sent a total of 2657 questionnaires to them. The sample size was adequate. The survey was conducted during November 2017. Overall, 1791 nurses responded (response rate = 67.4%). After excluding incomplete questionnaires, 1716 fully completed questionnaires were analysed.

### Data analysis

For the preliminary analysis, descriptive statistics concerning participants’ demographic characteristics were performed. Skewness < |2| and kurtosis < |7| were calculated to indicate normality, and items that did not meet these criteria were excluded from the analyses (Kim 2013). We also excluded items that met ceiling effect, floor effect and item‐total (I‐T) correlation < 0.2 criteria.

The construct validity was assessed through an exploratory factor analysis (EFA), confirmatory factor analysis (CFA), criterion‐related validity and concurrent validity. Prior to the EFA, we performed Bartlett’s sphericity test for factor analysis compatibility and calculated the Kaiser–Meyer–Olkin (KMO) measure of sampling adequacy to determine the sample validity. In the EFA, the number of factors was determined by retaining all factors with an eigenvalue of 1, and items with factor loadings over 0.4 were retained. The factors extracted from the EFA were carefully interpreted and named. A CFA was then implemented to confirm whether findings of the EFA were consistent with the theoretical model of the PASN‐J.

Part of the nurse’s professional behaviour scale was used for the criterion‐related validity. PASN‐J measures the professional attitudes of nurses at the cognitive level. Because cognition precedes behaviour (Kraus 1995), nurses with high levels of cognition for professionalism are presumed to have high levels of professional behaviour. Professional behaviour was measured using the four‐item career development subscale of the professional behaviour scale developed by Sawada (2009). This subscale is a five‐point Likert‐type scale, consisting of items such as ‘I regularly read professional journals related to work’ and ‘I pursue personal development as a nurse’. A high score provided a strong indication of engagement in career development behaviour (i.e., a higher degree of professionalism at the behavioural level). The reliability and construct validity were verified by the developer, and the Cronbach’s α in this survey was 0.86.

Nursing practical ability was used for concurrent validity. Professional nursing attitudes are related to self‐learning behaviour; the more active self‐learning behaviours are, the higher the ability to practice nursing. PASN‐J score and nursing practice ability are considered theoretically correlated. For the measurement of nursing practice ability, we used a seven‐item measure of concrete judgment ability – a part of an instrument used to measure professional autonomy in nursing (Kikuchi & Harada, 1997). One example item was ‘I can comprehend patient needs and select appropriate nursing care’. Respondents answered each item on a five‐point Likert scale (5 = strongly agree; 1 = strongly disagree); higher scores indicated greater concrete judgment ability. The Cronbach’s α in this survey was 0.90.

In addition, focusing on level of education and level of expertise, the known‐groups validity was verified. In Japan, nurses who completed over six months of advanced professional education are considered certified nurses (CNs). Because of the motivation required, we hypothesized that nurses with master’s/doctoral degrees or who were CNs would display higher professional attitudes scores.

The reliability of PANS‐J was assessed by the internal consistency. Cronbach’s *α* for each factor was confirmed. Statistics were calculated using SPSS Statistics version 23 (IBM Corp., Armonk, NY, USA).

### Ethical considerations

In accordance with the ethical guidelines for medical and health research involving human participants in Japan and the 1995 Helsinki Declaration (as revised in Edinburgh 2000), we conducted this study with approval from the Clinical Research Ethics Committee of Tohoku University Graduate School of Medicine (approval number 2016‐1‐483). We fully explained the purpose of the research and the contents to the nurse executives of each facility, who were asked not to pressure nurses to participate. When requesting individual nurses to respond to the questionnaire, we explained the purpose of the study, that participation was voluntary, and that their responses would remain confidential. Returning the questionnaire was interpreted as consent to participate. All questionnaires were completed anonymously.

## Results

### Participants’ demographics (n = 1716)

Most participating nurses were female (93.1%). The mean age was 36.3 years (SD = 10.4), and ages ranged from 21 to 69 years. The mean number of years working as a nurse was 13.6 (SD = 10.0), with a range from 1 to 48 years (Appendix 3).

### Item analysis

The descriptive statistics for each item included the range, mean, standard deviation, skewness and kurtosis, shown in Table 1. All items met the normality criteria. The ceiling effect occurred in 12 items (No. 1, 3, 8, 22, 23, 24, 25, 26, 28, 29, 30 and 33), and the I‐T correlation was less than 0.2 in two items (No. 57 and 58). The factor structure of PASN‐J was presumed by EFA with 45 items excluding these 14 items.

**Table 1:** Item descriptive statistics

	Range	Mean	SD	Skewness	Kurtosis	Item‐total correlation
Item1	1–5	4.59	0.62	−1.46	2.09	0.36**
Item2	1–5	4.20	0.78	−0.64	−0.21	0.44**
Item3	1–5	4.17	0.85	−0.90	0.56	0.46**
Item4	1–5	4.00	0.90	−0.66	0.05	0.57**
Item5	1–5	3.93	0.87	−0.52	−0.10	0.52**
Item6	1–5	3.70	0.98	−0.54	−0.03	0.57**
Item7	1–5	3.95	0.89	−0.73	0.53	0.53**
Item8	1–5	4.35	0.74	−1.21	2.14	0.47**
Item9	1–5	3.73	1.00	−0.49	−0.23	0.55**
Item10	1–5	4.06	0.86	−0.77	0.41	0.55**
Item11	1–5	4.01	0.84	−0.63	0.15	0.50**
Item12	1–5	3.36	0.94	−0.05	−0.13	0.50**
Item13	1–5	3.47	0.91	−0.16	−0.06	0.55**
Item14	1–5	3.57	0.90	−0.17	−0.07	0.58**
Item15	1–5	3.14	1.14	0.01	−0.73	0.47**
Item16	1–5	3.46	1.21	−0.27	−0.85	0.52**
Item17	1–5	3.52	1.19	−0.36	−0.76	0.55**
Item18	1–5	3.07	1.15	0.06	−0.59	0.53**
Item19	1–5	2.40	1.04	0.46	−0.26	0.45**
Item20	1–5	3.45	1.12	−0.38	−0.60	0.52**
Item21	1–5	2.98	1.10	0.02	−0.48	0.55**
Item22	1–5	4.47	0.71	−1.21	1.00	0.37**
Item23	1–5	4.51	0.70	−1.50	2.83	0.37**
Item24	1–5	4.62	0.62	−1.82	4.37	0.31**
Item25	2–5	4.63	0.57	−1.42	1.68	0.38**
Item26	2–5	4.65	0.58	−1.62	2.49	0.32**
Item27	1–5	3.98	0.86	−0.51	−0.11	0.53**
Item28	1–5	4.46	0.67	−1.03	0.72	0.49**
Item29	1–5	4.33	0.73	−0.89	0.55	0.58**
Item30	1–5	4.34	0.69	−0.85	0.86	0.48**
Item31	1–5	4.17	0.78	−0.75	0.49	0.45**
Item32	1–5	4.28	0.70	−0.60	−0.12	0.51**
Item33	1–5	4.41	0.66	−0.82	0.28	0.44**
Item34	1–5	4.21	0.74	−0.62	−0.04	0.53**
Item35	1–5	4.10	0.82	−0.67	0.24	0.43**
Item36	1–5	3.83	0.89	−0.43	−0.08	0.50**
Item37	1–5	4.08	0.82	−0.74	0.47	0.45**
Item38	1–5	3.85	0.91	−0.67	0.33	0.45**
Item39	1–5	3.83	0.79	−0.27	−0.07	0.55**
Item40	1–5	3.67	0.89	−0.40	0.08	0.53**
Item41	1–5	3.44	0.98	−0.23	−0.21	0.52**
Item42	1–5	3.72	0.93	−0.45	−0.03	0.43**
Item43	1–5	3.94	0.85	−0.70	0.71	0.41**
Item44	1–5	2.80	1.14	0.15	−0.51	0.50**
Item45	1–5	2.70	1.05	0.19	−0.32	0.52**
Item46	1–5	3.01	1.13	0.02	−0.60	0.55**
Item47	1–5	3.23	1.11	−0.22	−0.51	0.57**
Item48	1–5	3.25	1.13	−0.21	−0.54	0.59**
Item49	1–5	3.36	1.04	−0.15	−0.39	0.50**
Item50	1–5	3.09	1.03	0.02	−0.31	0.38**
Item51	1–5	3.13	0.91	−0.04	0.25	0.47**
Item52	1–5	3.64	0.91	−0.53	0.34	0.51**
Item53	1–5	3.42	0.93	−0.19	0.06	0.52**
Item54	1–5	3.25	1.01	−0.17	−0.13	0.52**
Item55	1–5	3.76	0.95	−0.29	−0.53	0.27**
Item56	1–5	3.98	0.94	−0.63	−0.16	0.26**
Item57	1–5	3.14	1.10	0.09	−0.47	0.14**
Item58	1–5	3.32	1.13	−0.15	−0.70	0.10**
Item59	1–5	4.06	0.88	−0.78	0.46	0.34**

* *p* < .05.

** *p* < .01.

### Construct validity

The KMO measure of sampling adequacy was 0.921, and Bartlett’s test of sphericity was significant (*χ* ^2^ = 44956.0, df = 990, *P* < 0.01). An EFA using the maximum likelihood method with promax rotation was performed to examine the factor structure of the PASN‐J. Using the initial eigenvalue and screen plot as criteria, we extracted a total of eight factors. Seven items (No. 9, 10, 19, 27, 37, 38 and 39) with factor loadings of <0.4 were excluded from this eight‐factor structure (Table 2).

**Table 2:** Exploratory factor analysis of the 45 items

Item	Factor 1	Factor 2	Factor 3	Factor 4	Factor 5	Factor 6	Factor 7	Factor 8
Item16	0**.970**	−0.027	−0.028	−0.039	−0.026	−0.032	0.014	−0.013
Item17	0**.933**	0.004	−0.039	0.001	−0.021	−0.025	0.016	0.018
Item18	0**.823**	0.039	−0.007	0.016	−0.040	−0.003	0.002	−0.013
Item15	0**.719**	0.023	0.015	−0.021	−0.033	0.012	−0.003	−0.013
Item21	0**.566**	−0.006	0.060	0.036	0.113	0.056	−0.024	−0.003
Item20	0**.502**	−0.042	0.076	0.033	0.137	0.057	−0.046	0.045
Item51	0.036	**0.871**	−0.076	−0.017	−0.003	−0.013	−0.003	−0.052
Item50	−0.002	**0.827**	−0.077	−0.008	−0.008	−0.045	−0.034	−0.083
Item53	−0.020	**0.732**	0.017	0.069	−0.025	0.030	−0.019	0.069
Item54	0.017	**0.730**	0.030	0.009	0.001	0.019	−0.029	0.050
Item52	−0.045	**0.672**	0.050	0.013	0.008	0.062	−0.010	0.074
Item49	0.014	**0.655**	0.101	−0.059	0.025	−0.013	0.022	0.019
Item47	−0.006	−0.075	**0.973**	0.004	−0.009	−0.017	−0.049	0.030
Item46	−0.030	0.012	**0.879**	−0.016	−0.005	0.001	−0.059	0.024
Item48	0.032	−0.029	**0.846**	0.044	−0.055	0.041	−0.036	0.024
Item44	0.016	0.017	**0.713**	0.012	0.015	−0.075	0.032	−0.041
Item45	0.017	0.137	**0.600**	−0.056	−0.024	−0.006	0.121	−0.062
Item31	−0.021	0.004	0.008	**0.835**	−0.051	−0.018	−0.022	−0.035
Item32	0.002	−0.024	−0.012	**0.829**	−0.005	−0.024	0.037	0.047
Item34	0.041	−0.044	0.022	**0.741**	0.035	0.034	−0.009	0.015
Item36	0.013	0.029	0.016	**0.735**	0.033	0.026	−0.011	−0.080
Item35	−0.029	0.033	−0.024	**0.702**	−0.004	−0.016	0.023	0.026
Item6	0.012	−0.041	0.009	−0.023	**0.856**	0.034	−0.036	−0.026
Item5	−0.006	0.015	−0.060	0.000	**0.793**	−0.066	0.029	0.013
Item4	−0.021	0.018	0.039	−0.022	**0.764**	0.045	−0.038	−0.03
Item7	0.000	−0.057	0.019	−0.028	**0.724**	0.038	0.024	0.010
Item2	0.055	0.082	−0.101	0.090	**0.487**	−0.071	0.027	0.057
Item13	−0.003	−0.029	−0.023	−0.022	−0.053	**0.992**	0.026	−0.008
Item12	0.04	0.035	−0.067	−0.013	−0.031	**0.832**	0.007	−0.017
Item14	0.019	−0.007	0.038	−0.014	0.032	**0.831**	−0.008	0.010
Item11	−0.075	0.033	0.016	0.111	0.159	**0.480**	−0.016	0.005
Item42	−0.004	−0.047	−0.073	0.003	−0.035	0.012	**0.938**	0.017
Item43	−0.010	−0.064	−0.059	0.004	−0.014	0.000	**0.854**	0.038
Item40	0.027	0.060	0.122	0.055	0.047	0.029	**0.520**	−0.025
Item41	−0.020	0.145	0.181	−0.038	0.079	−0.020	**0.503**	−0.051
Item56	0.004	−0.044	−0.027	−0.011	−0.006	0.016	−0.011	**0.905**
Item55	−0.007	0.051	−0.005	−0.007	0.015	−0.039	−0.014	**0.735**
Item59	0.010	0.050	0.074	−0.005	0.008	0.006	0.115	**0.410**
Eigenvalue	9.31	4.12	2.48	1.71	1.69	1.28	1.25	1.04
Per cent of total variance explained	24.50	10.80	6.50	4.50	4.50	3.40	3.30	2.70
Cumulative per cent	24.50	35.30	41.90	46.40	50.80	54.20	57.50	60.20

Factor loads over 0.4 are shown in bold.

The eight factors accounted for 60.2% of the variance in professional attitudes and were identified as follows: (1) improve nursing education (six items), (2) autonomy as a professional organization (six items), (3) job‐related independence (five items), (4) responsibility to fulfil requests (five items), (5) construction of scientific nursing (five items), (6) vitalization of professional organization (four items), (7) autonomous clinical judgment (four items) and (8) rejection of the servant image (three items).

**Fig 1:**
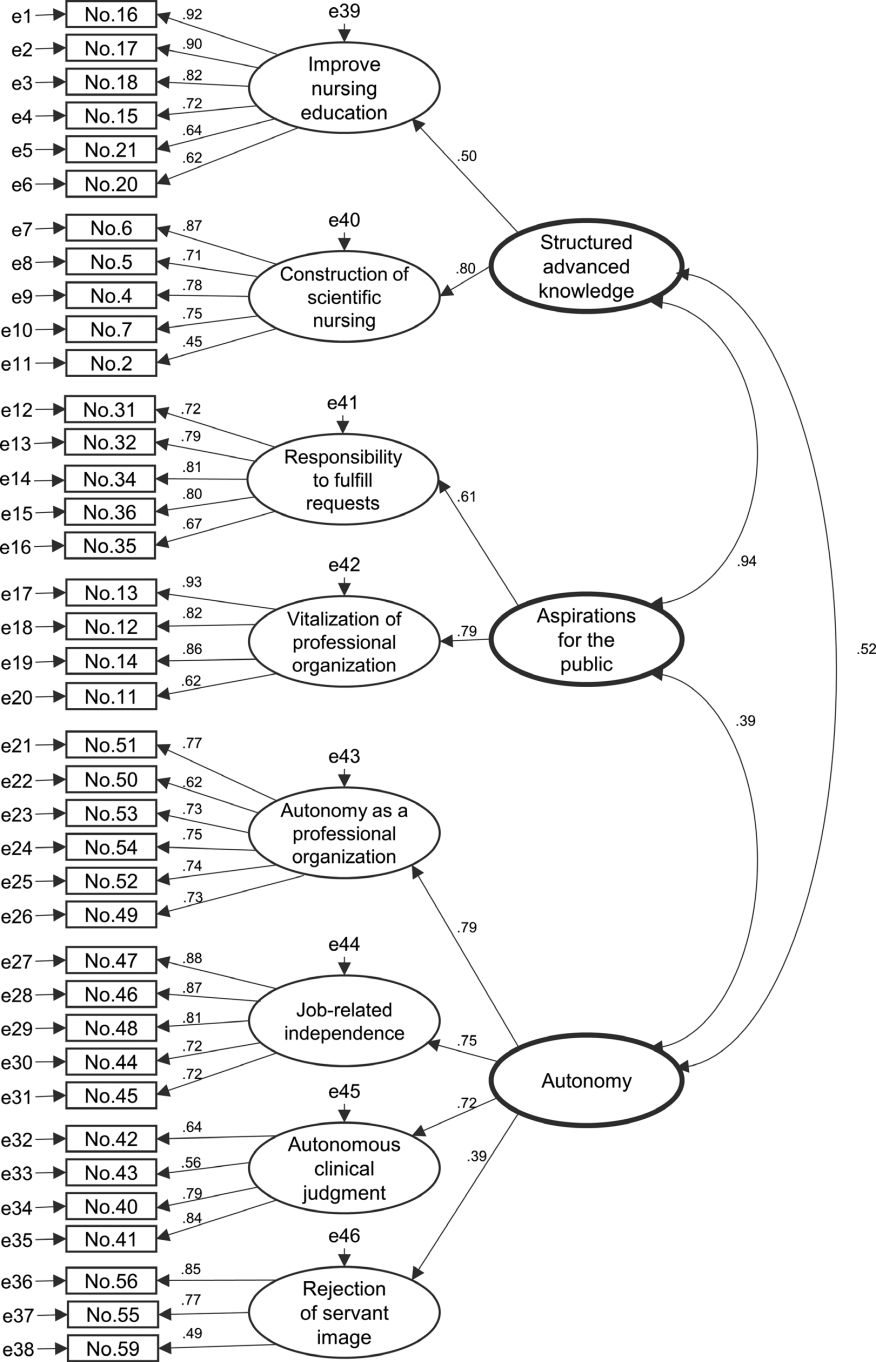
Confirmatory factor analysis of the Japanese version of the Professional Attitude Scale for Nurses.

A PASN‐J theoretical construct model that measures professional attitudes with three constructs in structured advanced knowledge, aspirations for the public and autonomy was verified by a CFA. Specifically, a CFA was conducted, assuming a hierarchical model in which the eight subconcepts identified by the EFA load onto the three key concepts shown in Figure 1. The fit indices for the hierarchical model were acceptable (*χ* ^2^ = 2223.4, df = 637, *P* < 0.001, goodness‐of‐fit index [GFI] = 0.935, adjusted GFI = 0.924, root mean square error of approximation = 0.038, comparative fit index = 0.959). Each item loaded significantly onto its corresponding first‐order factor, and each first‐order factor loaded significantly onto its second‐order factor.

### Criterion‐related validity, concurrent validity and known‐groups validity

The correlations among structured advanced knowledge, aspirations for the public and autonomy scores and the career development subscale were *r* = 0.347, 0.327 and 0.256, respectively (*P* < 0.001). The correlations among structured advanced knowledge, aspirations for the public and autonomy scores and nurses’ concrete judgment ability were *r* = 0.288, 0.271 and 0.425, respectively (*P* < 0.001; Appendix 4). The scores of nurses with a master’s/doctoral degree or CN qualifications tended to be higher than the scores of those without. The scores of nurses with master’s/doctoral degree or CN qualifications and the scores of others were as follows: structured advanced knowledge: 43.8 ± 7.4, 39.2 ± 7.7 (*P* < 0.001); aspirations for the public: 35.9 ± 5.6, 35.0 ± 5.3 (*P* = 0.15); autonomy: 67.7 ± 10.4, 61.1 ± 10.9 (*P* < 0.001; Appendix 5).

### Reliability

The reliability coefficients were evaluated after the CFA to assess whether the items of each of the three constructs measured the same construct. The Cronbach’s *α* was 0.882 for structured advanced knowledge, 0.875 for aspirations for the public and 0.904 for autonomy (Appendix 6).

## Discussion

The PASN‐J showed appropriate reliability and construct validity for assessing nurses’ professional attitudes at the cognitive level. The theoretical framework of the PASN‐J was based on the three traits considered necessary for professionals to embody – structured advanced knowledge, aspirations for the public and autonomy. We assumed a hierarchical model of the nurses’ professional attitudes. The hypothetical model was validated by exploratory and confirmatory factor analysis. Although a complex model was used, this study demonstrated that the data collected fit with a structure similar to the hypothetical model.

There was a significant positive correlation between the PANS‐J and professional behaviour. This finding is consistent with the premise of this research that cognition predicts future behaviour (Kraus 1995). The result of group comparisons revealed that participants who had obtained advanced nursing education had higher PANS‐J scores. There was also a correlation between the PANS‐J and nursing practical ability. The results of this study suggested that the PANS‐J scale has good validity and reliability in assessing nurses’ professional attitudes.

In this study, we presented a new tool for measuring professionalism of nurses. While scales had already been developed to measure nurses’ professional attitudes at the behavioural level, there was no appropriate scale to measure them at the cognitive level (Weis & Schank, 2009). PASN‐J enables us to examine this construct at the cognitive level outside of the restrictions of environmental constraints such as law, culture and institution. This is particularly necessary in countries such as Japan, in which nurses’ behaviours are highly constrained by their environments.

Another strength of this scale is that it was developed using the trait approach in consideration of the situation of modern nurses. The PASN‐J contained factors similar to those of existing scales, such as factors related to ethics, professional organization, autonomy and education (Hisar et al. 2010; Shohani & Zamanzadeh, 2017). These factors are important for modern nurses, but as social responsibility for nursing has been emphasized, more focus on the public role of nursing is needed (Stievano & Tschudin, 2019; Williams et al. 2016). The inclusion of the concept of aspirations for the public in the nurses’ professional attitudes was a novelty of PASN‐J. For example, ‘Responsibility to fulfil requests’ in aspirations for the public measured nurses’ attitudes in response to social requests and reflected the current situation in which nurses contribute to society using their knowledge and skills.

Additionally, ‘rejection of servant image’ was a crucial factor for nurses; however, it had not been mentioned before. Price & Hall, 2013; p 1504) analysed the history of the perception of nursing ‘in the context of recent career choice research and the need for contemporary images for nursing recruitment’. They noted that nurses are often presented as unscientific and less‐skilled helpers to physicians. To change these images of nursing, a strategy that emphasizes nurses’ knowledge and skills is necessary (Girvin et al. 2016; Price & Hall, 2013).

The results of this study supported the notion that nurses’ professional attitudes have a hierarchical structure. Goodness‐of‐fit scores were in the range that could be interpreted as a good model (Hooper et al. 2008). Additionally, the validity of the three‐factor structural model of the PASN‐J must be considered together with other validity evaluations. There was a reciprocal influence among PASN‐J scores, nurses’ practice ability and education level. The existence of this interrelationship is evidence of the validity of this instrument.

## Implications for nursing and health policy

The practical implications of using this scale include evaluating nursing education and promoting nurses’ professionalism, issues that need to be addressed particularly within Japan (Shibata 2017). By measuring the effect of education on professional attitudes and establishing a cycle of education and evaluation, this scale can be used to promote nurses’ professional growth. The PASN‐J will contribute to identifying the elements of undergraduate nursing education that require further emphasis. In addition, the PASN‐J could be used as evidence for developing nursing policies to promote the professional development of nurses. For example, graduate education and expertise education contribute to the improvement of systematic knowledge and autonomy but do not significantly contribute to the improvement of aspirations for the public. This study suggests the importance of advanced nursing education regarding the improvement of aspirations for the public. Moreover, it would still be interesting to compare the results of the PASN‐J and other measures of professional behaviour as a means of examining large disparities that may provide further evidence of environmental constraints. Ultimately, to evaluate nursing education with the PASN‐J enhances nurses’ professional attitudes and, as a result, improves the quality of nursing they provide, their nursing efficiency and patient outcomes.

### Limitations

This study had some key limitations. First, the sample only included hospital nurses. In the future, it is necessary to include not only hospital nurses but also nurses working in various other areas, such as nursing homes and visiting nurse agencies. Second, the results may be subject to social desirability bias. Since nurses are expected to behave as professionals, they may have responded in insincere but socially desirable ways. Other limitations include that this was a self‐report study and that we only examined reliability using Cronbach’s α coefficients.

## Conclusion

In this empirical study, we developed and validated a theory‐based scale to measure nurses’ professional attitudes. Our analysis demonstrated that the 38‐item PASN‐J, which contained three factors and eight subconcepts, has acceptable construct validity, concurrent validity and reliability. This tool can be used to measure the current state of nursing and to evaluate nursing education.

## Author contributions

Study design: NT, KA, SS

Data collection: NT

Data analysis: NT, KA, SS

Study supervision: KA

Manuscript writing: NT, KA, SS

Critical revisions for important intellectual content: NT.

## Supporting information

Supplementary Figure S1

Supplementary Tables S2–S6
