# Supplementary figures and images for: Developing and validating the Japanese version of professional attitude scale for nurses
Source: Int Nurs Rev. 2020 Oct 12;68(1):24–33. doi: 10.1111/inr.12627 (PMC8247416; doi:10.1111/inr.12627)

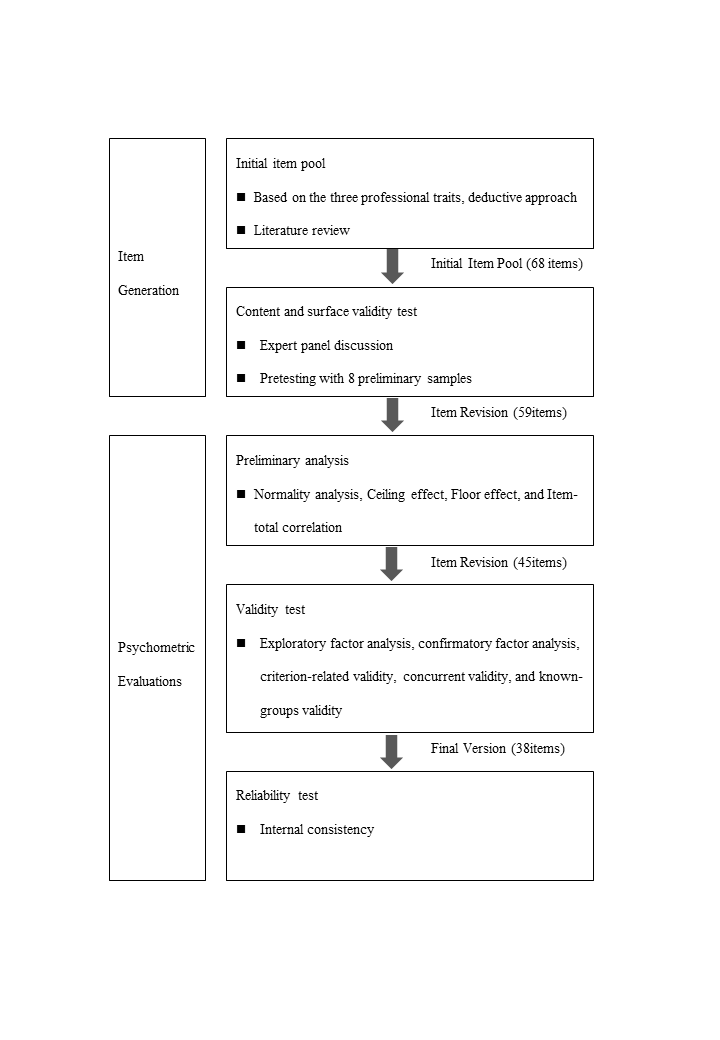

Supplement: Supplementary file 1 — Supplementary Figure S1 [file INR-68-24-s002.tif]
