# Supplementary material for: Developing and validating the Japanese version of professional attitude scale for nurses
Source: Int Nurs Rev. 2020 Oct 12;68(1):24–33. doi: 10.1111/inr.12627 (PMC8247416; doi:10.1111/inr.12627)
Supplement: Supplementary file 2 — Supplementary Tables S2–S6 [file INR-68-24-s001.docx]

Appendix 2. Examples of items

| Measurement concept | Examples of items |
| --- | --- |
| Systematic knowledge | I think that the education to obtain a nursing license is too short at only three years. |
|  | I think that nurses should conduct research to increase scientific knowledge about nursing. |
| Aspirations for the public | I think that nurses should not refuse to deliver patient care without a proper reason. |
|  | I think that the continued development of nursing is important to the unity of professional organizations. |
| Autonomy | I think that practicing nursing without a medical doctor’s supervision is desirable. |
|  | I think that nurses should be evaluated within the field of nursing rather than in other occupations. |

Appendix 3. Participants’ demographics (n = 1716)

| Variable | | | | n (%) |
| --- | --- | --- | --- | --- |
| Age (years) | | | | |
|  | ≥25 | | | 301 (17.5) |
|  | 26–35 | | | 594 (34.6) |
|  | 36–45 | | | 461 (26.9) |
|  | 46–55 | | | 274 (16.0) |
|  | >55 | | | 86 (5.0) |
|  | Range: 21–69 | | | Mean (SD): 36.3 (10.4) |
| Number of years working as a nurse | | | | |
|  | ≥5 | | | 476 (27.7) |
|  | 6–15 | | | 583 (34.0) |
|  | 16–25 | | | 412 (24.0) |
|  | >25 | | | 245 (14.3) |
|  | Range: 1–48 | | | Mean (SD): 13.6 (10.0) |
| Sex | | | | |
|  | Male | | | 118 (6.9) |
|  | Female | | | 1598 (93.1) |
| Position | | | | |
|  | | Nurse manager | | 125 (7.3) |
|  | | Assistant nurse manager | | 217 (12.6) |
|  | | General (Staff) | | 1363 (79.4) |
|  | | Other | | 11 (0.6) |
| Professional qualification | | | | |
|  | | | Certified nurses | 40 (2.3) |
| Education | | | | |
|  | Vocational school for nurses’ assistants followed by vocational school for registered nurses (two years) | | | 281 (16.4) |
|  | Vocational school or junior college for registered nurses (three years) | | | 893 (52.0) |
|  | Baccalaureate in nursing program (four years) | | | 485 (28.3) |
|  | Master’s program in nursing | | | 42 (2.5) |
|  | Other | | | 12 (0.7) |

**Appendix 4** The criterion-related validity and concurrent validity of the Japanese version of the Professional Attitude Scale for Nurses

|  | Professional behavior | Concrete judgement | |
| --- | --- | --- | --- |
|  | r | r | |
| Structured advanced knowledge | .347*** | .288*** | |
| Aspirations for the public | .327*** | .271*** | |
| Autonomy | .256*** | .425*** | |
| Pearson product-moment correlation coefficient　　　　　　　*p＜.05　**p＜.01　***p＜.001 | | |  |

**Appendix 5** Known-group validity of the Japanese version of the Professional Attitude Scale for Nurses

|  |  | Master’s/doctoral degree, CN  n=79 | Others  n=1637 | p |
| --- | --- | --- | --- | --- |
| Structured advanced knowledge | （11–55） | 43.8±7.4 | 39.2±7.7 | *** |
| Aspirations for the public | （9–45） | 35.9±5.6 | 35.0±5.3 | .15 |
| Autonomy | （18–90） | 67.7±10.4 | 61.1±10.9 | *** |
| Pearson product-moment correlation coefficient　　　　　　　*p＜.05　**p＜.01　***p＜.001 | | | | |

**Appendix 6** Reliability of the Japanese version of the Professional Attitude Scale for Nurses

|  | Cronbach’s α |
| --- | --- |
| Structured advanced knowledge | 0.882 |
| Aspirations for the public | 0.875 |
| Autonomy | 0.904 |
